# GNSS-ISE: Instruction Set Extension for GNSS Baseband Processing

**DOI:** 10.3390/s20020465

**Published:** 2020-01-14

**Authors:** Krzysztof Marcinek, Witold A. Pleskacz

**Affiliations:** 1 Warsaw University of Technology, Institute of Microelectronics and Optoelectronics, ul. Koszykowa 75, 00-662 Warsaw, Poland; 2 ChipCraft Sp. z o.o., 20-262 Lublin, Poland

**Keywords:** instruction set extension, ISE, multi-frequency, multi-constellation, GNSS receiver, software defined radio, SDR

## Abstract

This work presents the results of research toward designing an instruction set extension dedicated to Global Navigation Satellite System (GNSS) baseband processing. The paper describes the state-of-the-art techniques of GNSS receiver implementation. Their advantages and disadvantages are discussed. Against this background, a new versatile instruction set extension for GNSS baseband processing is presented. The authors introduce improved mechanisms for instruction set generation focused on multi-channel processing. The analytical approach used by the authors leads to the introduction of a GNSS-instruction set extension (ISE) for GNSS baseband processing. The developed GNSS-ISE is simulated extensively using PC software and field-programmable gate array (FPGA) emulation. Finally, the developed GNSS-ISE is incorporated into the first-in-the-world, according to the authors’ best knowledge, integrated, multi-frequency, and multi-constellation microcontroller with embedded flash memory. Additionally, this microcontroller may serve as an application processor, which is a unique feature. The presented results show the feasibility of implementing the GNSS-ISE into an embedded microprocessor system and its capability of performing baseband processing. The developed GNSS-ISE can be implemented in a wide range of applications including smart IoT (internet of things) devices or remote sensors, fostering the adaptation of multi-frequency and multi-constellation GNSS receivers to the low-cost consumer mass-market.

## 1. Introduction

The global GNSS (Global Navigation Satellite System) market has been growing over the recent years [1,2]. Location-based services (LBS) utilizing GNSS positioning have become a part of everyday life. Nowadays, the LBS consumer market is dominated by single-frequency GPS (US global positioning system) C/A (coarse acquisition signal) only and low-cost, highly integrated GNSS receivers [3,4,5,6,7,8,9]. However, low precision and low reliability are their main limitations. What is more, insufficient positioning in challenging environments, susceptibility to multipath interference, jamming, and spoofing further decrease the LBS segment coverage by such GNSS receivers. Such inconveniences are typically overcome by employing multi-constellation, multi-frequency receivers and by using additional complementary positioning technologies (i.e., inertial measurement unit—IMU) when necessary [10].

With the new GNSS signals transmitted at frequencies such as L2, E6, or L5/E5, analogue-receiving circuits capable of simultaneous reception of many navigational systems began to appear [11,12,13,14,15]. This fact increased the demand for flexible computing platforms capable of processing the received signals. Purely software-based solutions can easily keep up with the new reception algorithms and are able to handle multiple standards at once. On the other hand, the wide processing bandwidth, and, therefore, the high sampling rates, is a major challenge in the case of direct processing in a DSP (digital signal processing) processor, especially in the case of the low-budget mass market. Dedicated hardware units for GNSS baseband processing, therefore, seem indispensable. One such solution is the example of using an expansion card for a laptop [16]. It contains a programmable analogue front-end and a set of digital correlators capable of receiving both GPS, GLONASS (GLObal NAvigation Satellite System of Russian government), and GALILEO (European global satellite navigation system) signals. Digital signal processing is carried out using PC software. High efficiency and program flexibility are the biggest advantages of this solution, whereas the very low scale of integration is a disadvantage. Subsequent work transfers the correlator layer to the form of a dedicated peripheral module located on the internal microprocessor bus. The AGGA-4 (Advanced GPS/GLONASS Application-specific integrated circuit (ASIC)) receiver [14] is a dedicated GNSS receiver in the form of a system-on-module (SoM) for the space segment. The authors of the NAPA (NAvigation chip for Pedestrian navigation and higher-precision Applications) system approached the topic in a similar way [15]. The solution presented in [17] utilizes the system-on-chip (SoC) technique. In addition to the obvious advantages, the implementation of the GNSS block as a peripheral on the microprocessor bus is associated with limited bandwidth, as well as limited flexibility. There is also no possibility of adapting to new modulation techniques or advanced tracking algorithms such as ASPeCT (Autocorrelation Side-Peak Cancellation Technique) [18] or the TM61 technique [19]. The next step in the evolution of GNSS receivers was the adoption of the ASIP (application-specific instruction set processor) technique. It consists of a specialized processor adapted to the needs of a particular class of tasks. One of the examples of an ASIP system is a reconfigurable block supporting the calculation of position, velocity, and time—PVT [20]. As a result, the CORDIC (COordinate Rotation DIgital Computer) processor [21] for trigonometric calculations was developed. However, in this example, the ASIP technique has not been used to improve the time-critical blocks of correlators. They have been implemented in the form of a set of fixed, unconfigurable blocks. Focusing on the PVT computation places these works as a complementary to that presented in this paper, i.e., low-level baseband processing rather than competitive. On the other hand, the authors presented only simulation and field-programmable gate array (FPGA) results, while this work presents a GNSS-instruction set extension (ISE) implemented in the fully integrated multi-constellation and multi-frequency single-chip GNSS receiver ASIC (application-specific integrated circuit). The only attempt the authors of this publication are aware about to improve low-level GNSS processing using an instruction set extension was presented in [22]. However, in that publication, only correlation operations were taken into account. In addition, the author did not provide any results regarding the real impact of implemented instructions on the GNSS software receiver performance.

The goal of this work was to present a new and innovative approach to the topic of GNSS processor design. It involves the ASIP technique to develop a flexible GNSS baseband processing instruction set extension (ISE). This extension, applied to a general-purpose microprocessor system, defines a new, versatile GNSS processor architecture.

## 2. Instruction Set Extensions

**Figure 1:**
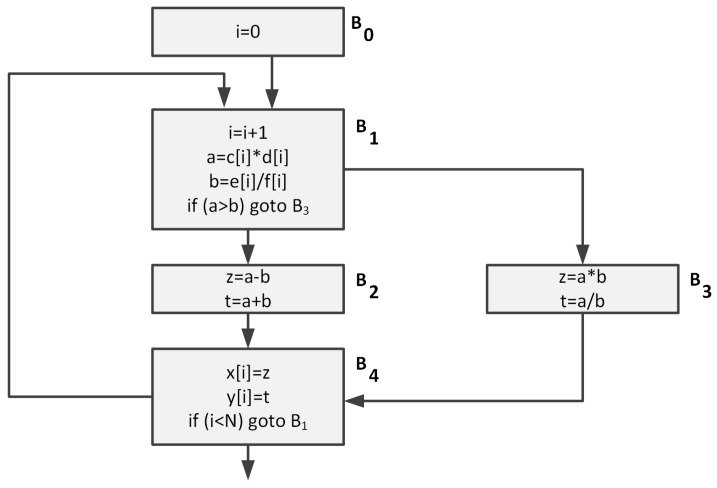
Control flow graph (CFG) example.

The need to develop new instruction set architectures (ISAs), modify existing ones, as well as create ISA extensions, results from the need to perform a certain group of tasks more quickly and without limiting the flexibility gained through the programmability. Although, for a long time, the task of identifying the potential candidates for new instructions rested with the system designers, in recent years, a number of methods for the automatic generation of instruction set extensions have been developed. They most commonly use compilers to transform an application written in a high-level language, such as C/C++, into the intermediate representation (IR) form, independent of the target processor’s ISA. The most common representation of the IR form is the control flow graph (CFG) [23]. Figure 1 shows the CFG of a sample application. Each node of the CFG graph represents the basic block of the application, while the edges represent the flow of control between the blocks.

**Figure 2:**
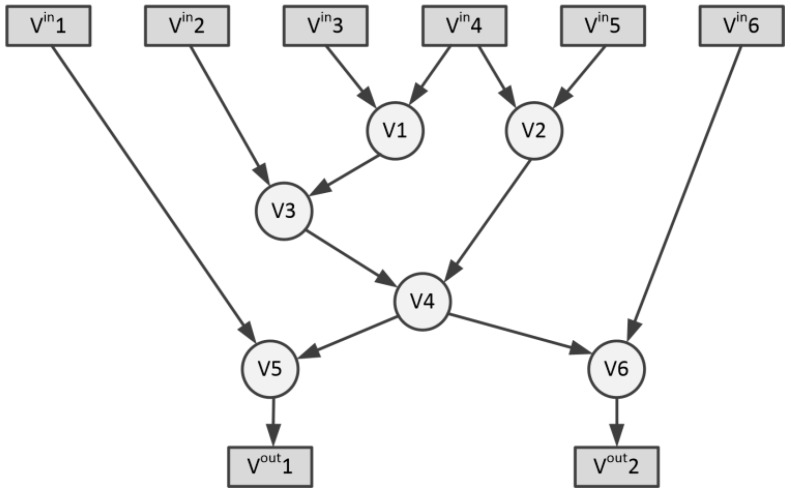
Basic block acyclic directed graph (DAG) example.

The basic blocks are sets of successive basic operations (such as addition, subtraction, multiplication, shifting, etc.) that do not contain the dependencies regarding the control flow. The individual basic blocks can be represented as acyclic directed graphs (DAG)—$G\left(V_{b}\cup V_{b}^{\mathrm{in}},E_{b}\cup E_{b}^{\mathrm{in}}\right)$, in which the vertices (denoted as $V_{b}$) represent basic operations, while the edges (denoted as $E_{b}$) are data relations between them (see Figure 2). The $V_{b}^{\mathrm{in}}$ vertices are basic block inputs, while the edges $E_{b}^{\mathrm{in}}$ join the input data with the $V_{b}$ operations. Vertices denoted as $V_{b}^{\mathrm{out}}\subseteq V_{b}$ represent outputs from the basic block. The task of the new instruction template is to unify a number of basic operations within a single functional block. The potential candidate for the new instruction is graph $T\left(V_{t}\cup V_{t}^{\mathrm{in}},E_{t}\cup E_{t}^{\mathrm{in}}\right)$, i.e., a sub-graph of the DAG graph G. $V_{t}$ vertices represent the basic operations contained in the new instruction template, while the $E_{t}$ edges are the data relationships between them. The $V_{t}^{\mathrm{in}}$ vertices are the inputs of the basic block. They may be the vertices of the G graph, or they may result from basic operations not included in the T template: $V_{t}^{\mathrm{in}}\subseteq V_{b}^{\mathrm{in}}\cup \left(V_{b}/V_{t}\right)$.

**Figure 3:**
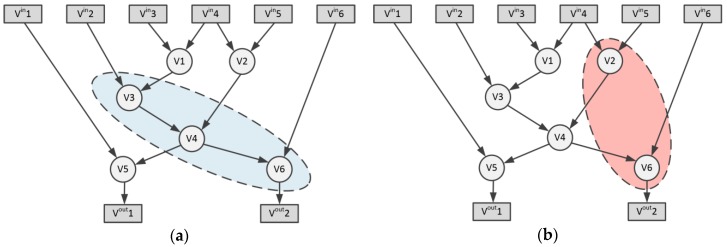
Vertices selection for new instruction template: (**a**) Convex graph selection for new instruction template; (**b**) Non-convex instruction template selection.

Graph convexity is an important concept in instruction set generation theory. The template T is convex if there is no path in graph G from node $u\in V_{t}$ to node $w\in V_{t}$ running through node $v\notin V_{t}$. The convexity of the graph T ensures that the graph G’ created after replacing the G template with a new custom operation (see Figure 3a) is acyclic. This is a condition that allows the basic block to be executed in the processor. Figure 3b shows the attempt to include V2 and V6 nodes into a new instruction template. This would cause the mutual dependence of unified nodes V2 and V6 on node V4, making the entire operation not feasible in the sequential execution of operations.

Methods of automatic generation of an instruction set extension have been studied extensively in recent years. Many algorithms have been developed, such as the MISO (multiple-input single-output) method or its extended MaxMISO version, which are based on partitioning the DAG graph into sub-graphs with the maximum number of inputs and one output [24]. Branch-and-bound optimization algorithms have been developed [25] that consist of the decomposition and controlled search of a set of acceptable solutions of a given problem. Paper [26] presents a solution based on the representation of the task of the automatic generation of instructions as an integer linear programming (ILP) problem. Subsequent work also addresses the accompanying problems, such as a limited number of input ports and the output file of the processor registers. Paper [27] proposed a method of serial access to inputs and outputs during the implementation of multi-cycle instructions. It has been shown, however, that additional input data can be obtained by redirecting from successive pipeline stages [28]. The solution used in the Tensilica Xtensa processor seems to be particularly interesting in this matter [29]. In order to increase the number of input and output operands, it uses an additional custom register file and instructions for transferring data between this file and the regular register file and memory. In almost all papers related to the subject matter, the results of the proposed algorithms are evaluated based on a narrow and repeatable set of input programs. These include, among others, cryptographic functions such as AES (advanced encryption standard), DES (data encryption standard), SHA (secure hash algorithms), and programs from the sets such as MiBench [30] and MediaBench [31]. Each of the mentioned algorithms also defines a measure of the quality of the proposed instructions and the objective function that forms a stop condition. The input arguments of the quality function are usually the parameters such as execution time reduction, reduction of power consumption, or increase in processor area, obtained from a comparison of the original solution and the application of the proposed instruction. It is worth noting here that a significant part of these parameters requires a precise definition of the applied semiconductor technology and the architecture of the tested system, which largely limits the universality of the obtained result. One of the measures of the evaluation of the generated candidates for instructions is also the comparison of results with templates obtained using the human designer analysis of optimized algorithms. It is worth mentioning that the results obtained by these methods are most often close to or identical to the commonly known solutions presented by human designers. This fact raises the question about the reason for such extensive research into the automatic synthesis of the list of orders. The most frequently mentioned explanations are the rapidly growing complexity of integrated circuits and their ever-shorter time to market. Automatic methods are used to significantly accelerate the design process of the system, as well as make the results independent of the experience of the designer. It cannot be overlooked, however, that they depend on the chosen method. In addition, the full context of application is not always known, e.g., in a general-purpose processor where the end-user decides how to use the hardware. The presence of an experienced designer, therefore, seems essential, making the described methods a valuable tool supporting the design process.

## 3. GNSS-ISE Development Method

The purpose of this work was to develop a universal GNSS instruction set extension. Universality is understood here in the functional and implementation scope. First, it is assumed that a purely software-based solution is a reference point, because it potentially enables the servicing of all current and future satellite navigation systems in any configuration, using any algorithms. In this respect, the functional versatility of the proposed instruction extension should avoid introducing restrictions on the selection of a supported navigation system and enable adaptation to new methods of its reception. Secondly, the universal instruction set extension should be independent of the processor architecture. In particular, despite the use of the same GNSS-ISE, the hardware implementation of the system supporting only a few L1 GPS channels will be different than a multi-frequency processor that simultaneously receives signals from GPS, GALILEO, GLONASS, etc.

In order to make the proposed extension independent of the processor architecture, the format of the instructions used in the currently most widespread RISC (reduced instruction set computing) processor architecture has been used. It operates with two input and one output values. On the other hand, the much older CISC (complex instruction set computing) architecture in today’s modern implementations is still implemented internally, mostly in the form of RISC-type micro-operation streams [32]. Parallel architectures such as VLIW (very long instruction word) or EPIC (explicitly parallel instruction computing) adopt RISC instructions without major modifications. In turn, more exotic architectures, such as the stack-based MISC (minimal instruction set computer) architecture, will require a greater involvement of the compiler or hardware units responsible for translating the developed instructions into their internal format. Furthermore, the functional versatility of the instruction set extension required the development of a new method, in which the previously described methods of automatic synthesis may or may not be used. The developed four-step method is described in the following sections.

### 3.1. Separation of Operations

A high-level CFG graph of the analyzed application is the input data to the proposed algorithm. For the purpose of this work, the authors would like to define the basic block as a sequence of high-level operations: $B_{i}=\left\{a_{0}^{i},a_{1}^{i},\ldots ,a_{k}^{i}\right\}$. Next, each of the basic block operations is a sequence of the basic operations: $a_{k}^{i}=\left\{a_{k_{0}}^{i},a_{k_{1}}^{i},\ldots ,a_{k_{n}}^{i}\right\}$. Defining high-level operations (e.g., phase-locked loop—PLL and delayed-locked loop—DLL, discriminator or loop filter in GNSS tracking loop) facilitates the understanding of the authors’ intentions while presenting this method. The first step of the proposed method is to identify the sub-sequences within individual operations in basic blocks that violate the functional versatility condition. For the purpose of this work, the authors formulated a $R_{use}\left(B,a_{k}\right)$ relation expressing the statement that the $a_{k}$ operation is contained in the basic block B instruction template. Consequently, the authors could propose the following relationship of the functional versatility, also called the condition of functional versatility:

(1) $$\begin{array}{c} R_{univ}\left(B,a_{k}\right)=∄\left\{a_{k_{m}}\right\}\left(\left\{a_{k_{m}}\right\}\in a_{k}\wedge a_{k}\in B\wedge \{a_{k_{m}}\}<a_{k}\right)\\ \left(R_{use}\left(B,a_{k}\right)R_{use}\left(B^{\prime },a_{k}\right)\right). \end{array}$$

It means that the condition of functional universality is violated if there is a sequence of basic operations $\left\{a_{k_{m}},a_{k_{m+1}},\ldots ,a_{k_{l}}\right\}$, being a sub-sequence of the operation $a_{k}$ and, in particular, not an $a_{k}$ operation, which, due to the fact that the operation $a_{k}$ is contained in the basic block B, operation $a_{k}$ cannot be contained in basic block B’, created by changing the parameters of the analyzed problem, e.g., due to appearance of a new GNSS system or improved tracking algorithm. In this case, the $a_{k}$ operation should be broken up so that the identified sub-sequences $\left\{a_{k_{m}}^{i}\right\}$ become a new operation in the basic block.

### 3.2. Prevention of Operations Merging

In the second step, similar to the previous one, all the $a_{k}$ operations that violate the $R_{univ}$ relation should be identified. The condition of functional universality is met by all the operations identified as $\left\{a_{k_{m}}^{i}\right\}$ sub-sequences in the previous step. The CFG graph of a GNSS receiver should include a frequency-locked loop—FLL, PLL/DLL discriminators and filters, as well as code generators, as separate basic blocks and prevent them from merging. This will ensure receiver implementation flexibility in terms of tracking loop architecture, filter structure, and external aiding.

### 3.3. Merging of Operations

The third step of the proposed method is the process of merging operations in the basic blocks. The automatic, as well as manual, instruction set generation algorithms’ task is to propose a new instruction template out of the basic block represented in the form of a DAG graph. The aim of this step is to obtain the DAG graphs with a large number of vertices. This will expand the search space, but, on the other hand, increase the probability of getting the highest-quality instruction template in terms of execution time reduction, reduction in power consumption, or increase in processor area. As in the previous steps, the merging process should take the $R_{univ}$ relation into account to maintain the developed instruction set extension flexibility.

### 3.4. Identification of Register Window

One of the most important problems while identifying new instruction templates is the large number of input and output vertices in DAG graphs. On the other hand, in many cases, input and output data of individual operations have local character. This means that the data are used only within these operations or in a predefined manner. Despite this, DAG graphs of such operations include them as full-fledged input and output nodes, significantly expanding the search space of automatic generation methods and limiting the quality of the obtained results. There are automatic methods of instruction generation, e.g., [33], which are able to extract nodes that can be realized as memory components visible from the perspective of the execution unit. The method proposed by the authors extends this solution to multi-channel applications. This method consists of the early identification of nodes having local character, their integration within a single register file, and their duplication to the form of windows, one per processed channel. At the time, only one window remains active, and its registers are permanently assigned as input and output nodes of individual execution units. As a result, one obtains execution units capable of holding their internal states (state-holding accelerator functional unit, AFU) [34] and able to switch between processed channels. In addition, this solution increases the number of available input and output operands of execution units and reduces the number of necessary memory references. Permanent assignation of individual registers to particular execution units reduces the complexity of the additional register file and, unlike its classic implementation, does not require additional address fields in the instructions format. Consequently, there is no need to modify the compiler.

### 3.5. Developed Method Algorithm

The algorithm of the proposed method is presented below (see Algorithm 1). It was used by the authors to develop a universal, multi-channel instruction set extension for GNSS baseband processing. The detailed description of the proposed instruction set is presented in the following sections.
**Algorithm 1.** GNSS-ISE development algorithm.1:Input: G, simplified application CFG graph
2:**for**B**in**G**do**
3: 
**for**
$a_{k}$
**in**
B
**do**

4:  **while**
$\neg R_{univ}\left(B,a_{k}\right)$
**do**▷ S1. Separation of Operations5:   Separate $a_{k}^{\prime }=\left\{a_{k_{m}}\right\}$ from $a_{k}$
6:   Add $a_{k}^{\prime }$ to set {marked}
7:  **end while**
8:  **if**
$\neg R_{use}\left(B^{\prime },a_{k}\right)$ then▷ S2. Prevention of Operations Merging9:   Add $a_{k}$ to set {marked}

10:  **end if**

11: **end for**
12: **for**
$\left\{a_{k},\ldots ,a_{k+l}\right\}$; $a_{n}\notin \left\{marked\right\}$
**in**
B
**do**▷ S3. Merging of Operations13:  **if**
$R_{univ}(B,\{a_{k}^{i},\ldots ,a_{k+l}^{i}\})$ then
14:   Merge operations
15:  **end if**

16: **end for**
17: **for**
$a_{k_{m}}$
**in**
B
**do**
▷ S4. Identification of Register Windows18:  Identify register windows
19: **end for**
20: Generate and evaluate instruction templates
21:**end for**


## 4. GNSS-ISE Instructions

The CFG graph of the GNSS multi-channel tracking loop prepared initially was processed through the proposed four-step algorithm. As a result, the authors obtained a number of DAG graphs that were manually processed, resulting in the GNSS-ISE instruction templates. The description of the instruction selection process is described in the following sections.

### 4.1. Carrier/Code Removal and Accumulation

**Figure 4:**
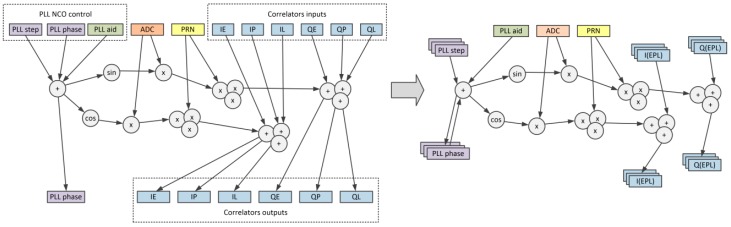
Identification of local variables to form custom register file windows.

**Figure 5:**
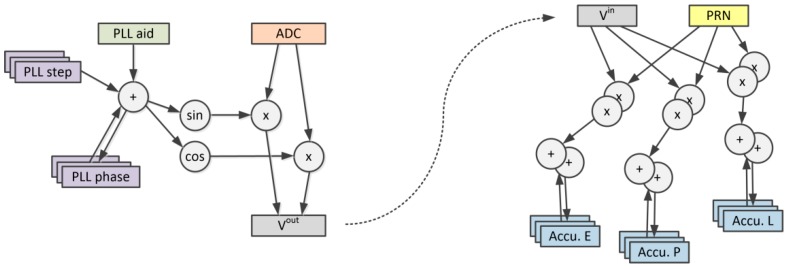
Extracting two instruction templates from DAG graph.

Figure 4 presents the process of identifying the variables that can be realized in form of custom register file windows. A DAG graph of the high-speed part of the GNSS tracking loop CFG contains carrier removal, spreading sequence removal, and data accumulation in in-phase (I) and quadrature (Q) early (IE, QE), and prompt (IP, QP) and late (IL, QL) branches. As many as eleven inputs contain the analog-to-digital converter’s sample (ADC), spreading sequence data (pseudo random noise, PRN), accumulator data (IE, IP, IL, QE, QP, QL), and PLL control inputs (current PLL phase and step). Seven outputs provide new correlators and PLL data. On the other hand, the DAG body contains only a few node layers. Using the traditional method of instruction set generation would result in many relatively simple instructions that would have questionable performance. One must remember that the new instruction template should replace a portion of the DAG that is big enough for its performance improvement to overcome the overhead of adding this template to the base processor ISA. After identifying the nodes that have local character and can be arranged in custom multi-channel register windows, the DAG graph was reduced to only three inputs and one output. Such a DAG graph can be easily divided into two new instructions—*gnss.carr.rem* for carrier removal and *gnss.accu.add* for code removal and accumulation (see Figure 5). For clarity, the *gnss.accu.add* instruction presented in Figure 5 has only three correlation branches—early (E), prompt (P), and late (L). The actual number of branches is implementation-dependent and can be five or even higher. The additional correlation branches can be used with methods like bump-jumping [35], and more sophisticated tracking algorithms like ASPeCT [18] or the TM61 [19] method for tracking the binary offset carrier (BOC) or time-multiplexed BOC (TMBOC) signals.

### 4.2. PLL/FLL/DLL Filter

**Figure 6:**
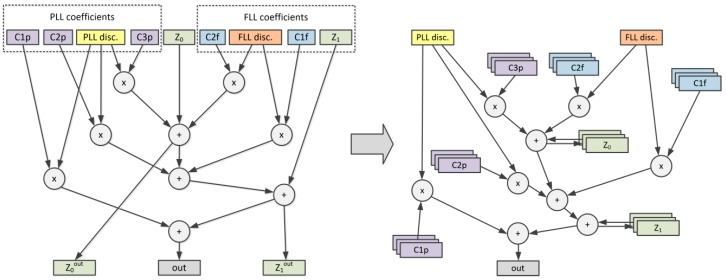
Identification of local variables in phase-locked loop (PLL) loop filter DAG.

Another example of using custom register file windows concerns the filter used for the phase-locked loop (PLL), frequency-locked loop (FLL), or delay-locked loop (DLL). Figure 6 presents a DAG representation of a third-order PLL filter with second-order FLL aiding from [36]. Nodes Z_0_ and Z_1_ present the two internal filter states that serve as inputs and outputs to be stored after the new filter value calculation. After the identification of local variables, the DAG graph can be reduced to have only two inputs and one output. This easily fits a single instruction template—*gnss.pll.flt*.

### 4.3. Carrier Discriminator

This and the next subsections present the parts of the GNSS tracking loop blocks and operations that were separated or prevented from merging based on the $R_{univ}\left(B,a_{k}\right)$ relation. The carrier discriminator is implementation-dependent. The GNSS carrier tracking loop can be realized as PLL or FLL. On the other hand, there are many implementations of the PLL discriminator itself. Therefore, the authors introduced the instruction templates—*gnss.pll.disc* and *gnss.pll.cost*, without specifying the architecture or implementation. However, the recommended choice is to use atan2(Q,I) as the PLL discriminator and $atan\left(\frac{Q}{I}\right)$ as the Costas PLL discriminator for their performance [36]. Moreover, the atan2(Q,I) function can also be used for atan2(cross,dot) calculation, which is an FLL discriminator.

### 4.4. Code Discriminator

**Figure 7:**
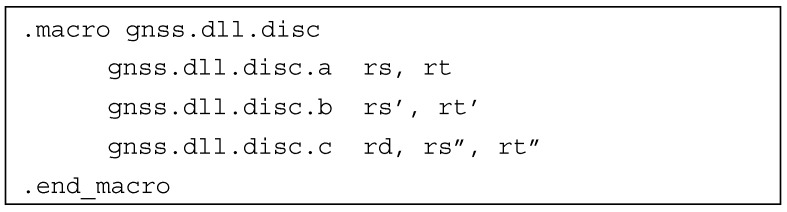
Code discriminator instruction macro.

Similar to the carrier discriminator, the code discriminator is implementation-dependent. In the literature, one can find a large number of DLL discriminator architectures, starting from the simple coherent $I_{E}-I_{L}$, through high-performance non-coherent $\frac{\left(I_{E}^{2}-I_{L}^{2}\right)+\left(Q_{E}^{2}-Q_{L}^{2}\right)}{\left(I_{E}^{2}+I_{L}^{2}\right)+\left(Q_{E}^{2}+Q_{L}^{2}\right)}$, to sophisticated discriminators utilizing many correlation branches [37]. Therefore, the authors would like to introduce a single instruction to perform the code discriminator function—*gnss.dll.disc*. As can be seen, the number of input parameters used can vary from two to six and more. This can be achieved by defining *gnss.dll.disc* as a macro-instruction, as presented in Figure 7. This approach is similar to the one used by the *MULScc* instruction in SPARC V8 (Scalable Processor ARChitecture) architecture [38].

### 4.5. Code Generation

The current GNSS system modulation techniques start from the most simple binary phase-shift keying (BPSK) modulation in GPS C/A, through more complex quadrature phase-shift keying (QPSK), many implementations of BOCs, multiplexed BOC (MBOC), composite BOC (CBOC), or TMBOC, to the most complex alternative BOC (AltBOC) modulation used in the GALILEO E5 band. Therefore, there is no trivial way to introduce universal hardware for all existing GNSS systems. On the other hand, there is a precedence of the GALILEO E1 signal, the spreading codes of which are memory-based. Therefore, the authors would like to recommend a memory-based PRN code generator with one mandatory instruction—*gnss.code.get*, used to get the next code sample. This approach allows all possible modulation techniques to be covered. The drawback is the necessity to implement a number of custom, additional, and windowed registers to enter the code generator parameters. For example, configuration registers would be used to enter primary and secondary PRN codes, set the carrier and code NCOs (numerically controlled oscillators), include BOC modulation, and configure branch spacing. The hardware complexity of code generation hardware is implementation-dependent. For example, there is no need to fully support the AltBOC modulation in low-cost GNSS receivers. On the other hand, the AltBOC modulation can be achieved using the software combination of two BOC streams.

### 4.6. Supplementary Instructions

**Figure 8:**
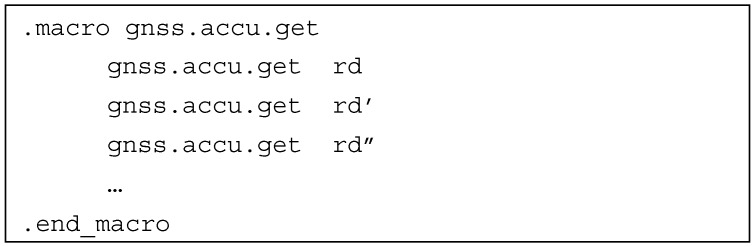
Accumulator data readout macro-instruction.

As shown in the previous sections, many of the former input and output nodes of the basic block DAGs were obtained in the form of custom, windowed register files. As each register window corresponds to one processing channel, the authors introduced instructions manipulating the channel index (*gnss.chann.set*, *gnss.chann.get*, *gnss.chann.incr*) to switch between the processed channels. The access to register windows can be easily implemented by adding two instructions, performing read and write access to the custom register file. For convenience, the register file access instructions were assigned with separate assembly mnemonics to free the programmer from the obligation to know the exact location of the interesting configuration register in the register file. As a result, without additional hardware, we obtained instructions capable of entering the numerically controlled oscillator (NCO) frequency (*gnss.carr.freq*, *gnss.code.nco.freq*, etc.), controlling loop filters (*gnss.pll.flt.rst*, *gnss.pll.flt.coef*, etc.), obtaining pseudo-range data—*gnss.code.rng*, or another macro-instruction for accumulator data readout (see Figure 8).

### 4.7. Special Instructions

The proposed GNSS-ISE was implemented and tested using simulations and FPGA implementations in [39]. The next verification step was to prepare the ASIC implementation of the multi-frequency GNSS receiver under the NaviSoC project [40]. During the design of the GNSS-ISE, the authors suspected that, despite the performance gain over the purely software-based solution, the GNSS receiver utilizing the introduced extension would still require a processor (CPU) with high computational power, similar to other existing software-defined radio (SDR) solutions. This is due to the fact that the processor is still involved in processing every ADC sample. With low-cost technology process nodes (~100 nm), it is hard to achieve a processor operation frequency significantly above 100 MHz. Consequently, with a high ADC sampling rate (~40 MHz), there is no time left to perform a pure SDR implementation. This motivated the authors to introduce new high-level GNSS-ISE instructions that can, besides the full SDR mode, place the GNSS engine in the semi-SDR and highly autonomous mode.

The *gnss.free.accu* instruction configures the desired GNSS channel to autonomously perform the {*gnss.carr.rem*, *gnss.code.get*, *gnss.accu.add*} instructions sequence every time a new ADC sample event occurs, without additional processor involvement. The CPU is only informed about the elapsing of the integration period event by means of an interrupt. This significantly relieves the processor as, now, the CPU reaction is needed every 1 ms for each channel in GPS C/A in bit synchronization mode or even every 20 ms while integrating over one GPS C/A bit duration. What is worth noting is that each channel can be configured to work with a different analog front-end, as each resulting channel processes, in parallel, samples from many ADCs. This is essential to perform multi-frequency and multi-constellation navigation.

The *gnss.free.update* instruction is used for further automation of the GNSS tracking loop. Together with the *gnss.free.accu* instruction, it performs the instruction sequence {*gnss.accu.get*, *gnss.pll.cost*, *gnss.pll.flt*, *gnss.dll.disc*, *gnss.dll.flt*, *gnss.carr.disc*, *gnss.code.disc*} for tracking loop update.

The *gnss.track.step* instruction is mainly used for testing and debugging the GNSS receiver working in pure SDR mode, semi-SDR mode, and full-automatic mode. The instruction emulates the ADC sample event and, consequently, allows for detailed insight into the tracking loop state at any time point.

Each of abovementioned *gnss.free.accu*, *gnss.free.update*, and *gnss.track.step* instructions may seem to limit the SDR capabilities as data processing is performed, in part, independently to the processor’s core. On the other hand, each of these modes can be stopped at any time or at any processing step, when the processor core decides that additional attention to tracking channels is needed. This feature can be particularly useful in a serial search acquisition algorithm as channels can be quickly reconfigured to, e.g., different Doppler frequencies, and accumulate data for a desired amount of time. The key factor to achieve high performance in this matter is to arrange GNSS hardware as a tightly coupled coprocessor extending the processor’s core base ISA, as presented in this paper. As described in the Introduction, GNSS hardware in the form of a peripheral block would face the limited bandwidth, especially in multicore systems, associated with the connection to a shared common system bus.

### 4.8. Instructions Summary

Table 1 summarizes the implemented GNSS-ISE instructions. The proposed instruction set extension was incorporated into the GCC toolchain in the form of a patch to the CC100-C processor [41].

## 5. GNSS-ISE Synthesis Results

**Figure 9:**
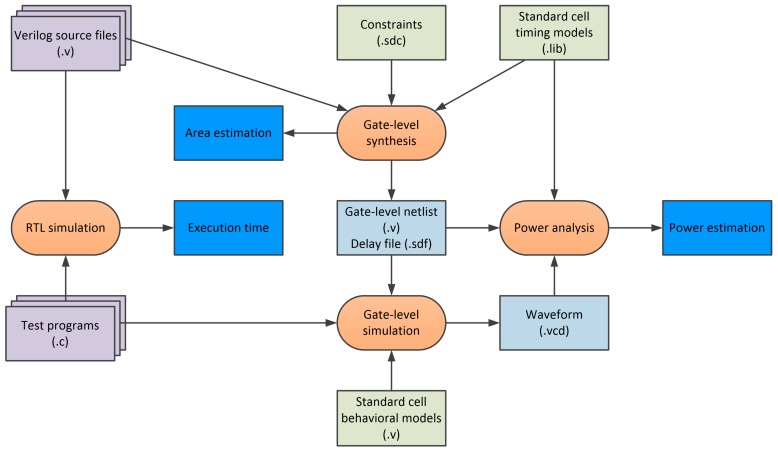
Execution time, area estimation, and power estimation flow.

This section presents the synthesis results of the GNSS navigation system containing four core processor (classic six-stage pipeline RISC architecture) and GNSS tracking channels in total number starting from zero to forty-eight. For this purpose, the whole GNSS navigation system incorporating a wide range of peripherals and 512 KiB of embedded memory was configured and synthesized for 100 MHz with the CMOS (complementary metal-oxide semiconductor) 130 nm process [39]. Figure 9 shows the used flow to obtain particular results. The tracking loop algorithms execution time is derived from the RTL (register-transfer-level) simulation. Gate-level synthesis of the GNSS navigation system with a different number of tracking channels was used to obtain the area estimation. Finally, gate-level simulation of the tracking loop algorithms enabled power consumption estimation.

**Figure 10:**
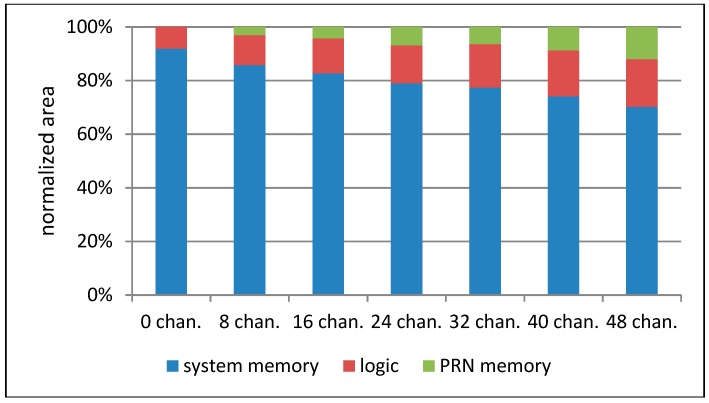
Normalized standard cells’ area as a function of GNSS tracking channels.

**Figure 11:**
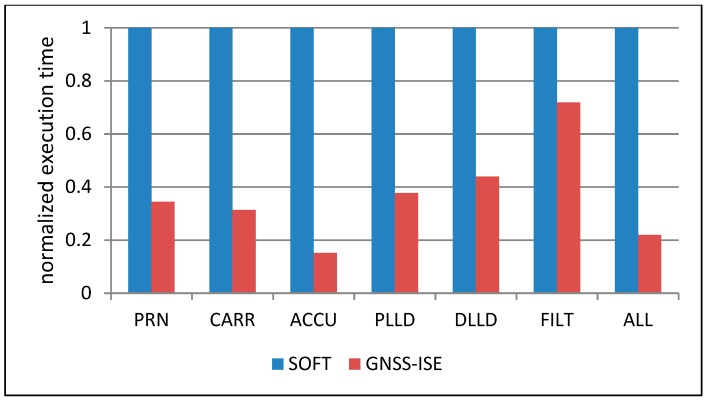
Normalized execution time of tracking loop tasks with GNSS-ISE and without GNSS-ISE.

**Figure 12:**
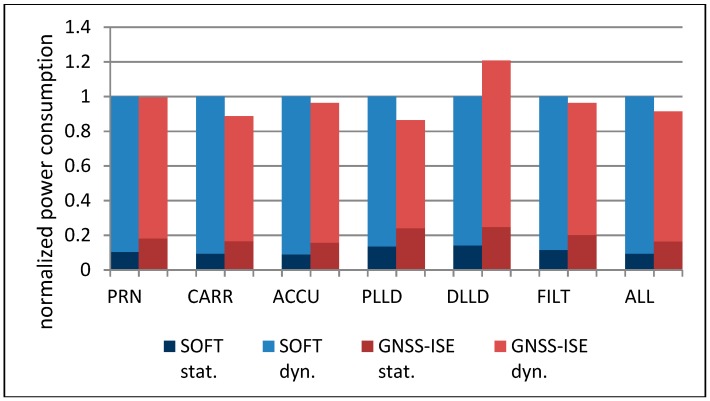
Normalized power consumption during execution of tracking loop tasks with GNSS-ISE and without GNSS-ISE.

**Figure 13:**
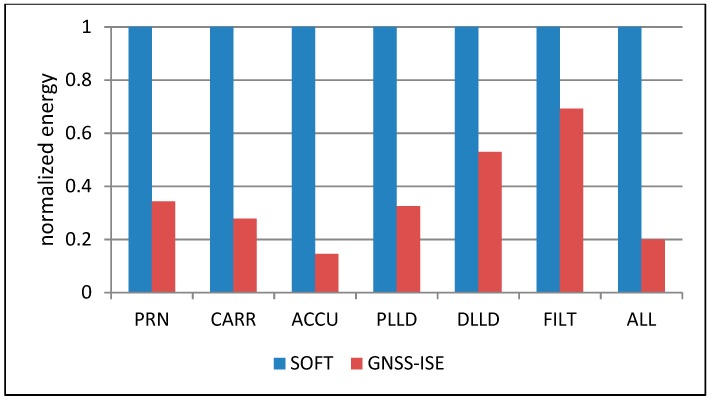
Normalized energy consumption during execution of tracking loop tasks with GNSS-ISE and without GNSS-ISE.

Figure 10 shows that with the growing number of tracking channels, the cells’ area grows linearly, but the most area-consuming part of the navigation system is still the large amount of embedded RAM memories. Figure 11 presents the comparison of the normalized execution time of particular tracking loop parts and the whole tracking loop. The executed algorithms include the spreading sequence generation (PRN), carrier removal (CARR), spreading sequence removal and accumulation (ACCU), PLL discriminator (PLLD), DLL discriminator (DLL), and loop filter (FILT), as well as a complete tracking loop (ALL). What could be expected is that the hardware implementation of particular GNSS tasks resulted in a significant execution time reduction. Please note that many of the GNSS-ISE aspects are implementation-dependent and the presented example shows only one of the possible implementations. The obtained results can differ among other implementations while the execution time reduction should still be observed. Figure 12 shows the normalized power consumption during execution of particular tracking loop parts and the whole tracking loop. What can be seen is that the power consumption changed insignificantly. Despite the processor core being relieved from processing GNSS data, the power consumption was caused by the additional hardware that was involved in the tracking loop execution. Figure 13 shows that with the maintained power consumption, but achieving significant time reduction, the energy needed for the execution of particular tracking loop parts and the whole tracking loop was also reduced significantly.

## 6. ASIC Hardware Results

**Figure 14:**
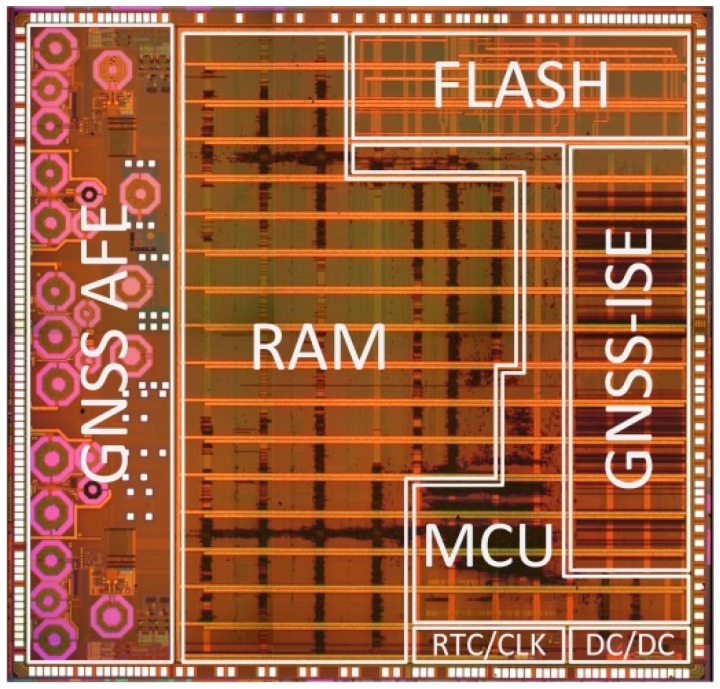
Microphotograph of CCNV1-A1, integrated multi-constellation, and multi-frequency GNSS receiver.

**Figure 15:**
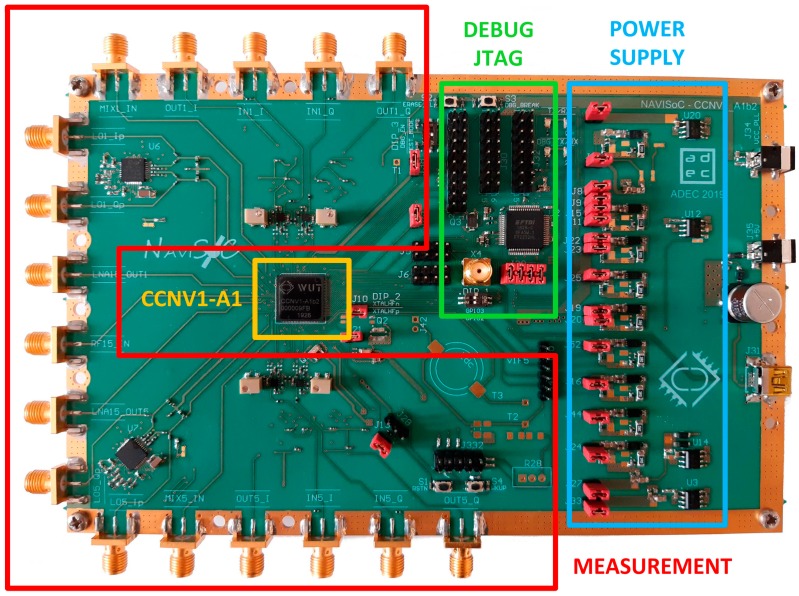
CCNV1-A1 measurement and evaluation board. The PCB (printed circuit board) board contains an CCNV1-A1 integrated circuit (IC) with surrounding radio frequency (RF) test and measurement infrastructure. The power supply is composed of a separate linear regulator for each analog and digital IC part for test purposes.

As stated before, ASIC implementation of the GNSS-ISE with the CC100-C [41] processor core was performed under the NaviSoC project [40]. Figure 14 presents a microphotograph of a 5.7 mm × 6.0 mm silicon die of the developed in [40] CCNV1-A1 navigation processor fabricated in the 110 nm eFlash process. Figure 15 presents the developed measurement and evaluation board. The chip consists of two analog front-ends for the L1/E1 and L5/E5 bands, a three-core microcontroller featuring a rich set of peripherals, 512 KiB SRAM (static random access memory), and 768 KiB eFlash. The peripherals associated with the GNSS receiver include two FFT-256 cores and a dedicated module supporting signal acquisition. The microcontroller peripherals include a number of communications interfaces (UARTs, SPIs, I2C, CAN, 1WIRE) and GPIO (general-purpose input/output), as well as timers, a watchdog, and a battery backed-up RTC (real-time clock) domain. Two out of the three cores support the GNSS-ISE instruction set extension for GNSS baseband processing. GNSS-ISE forms two autonomous 16-channel GNSS baseband coprocessors (32 channels in total). The third core is intended for user application.

**Figure 16:**
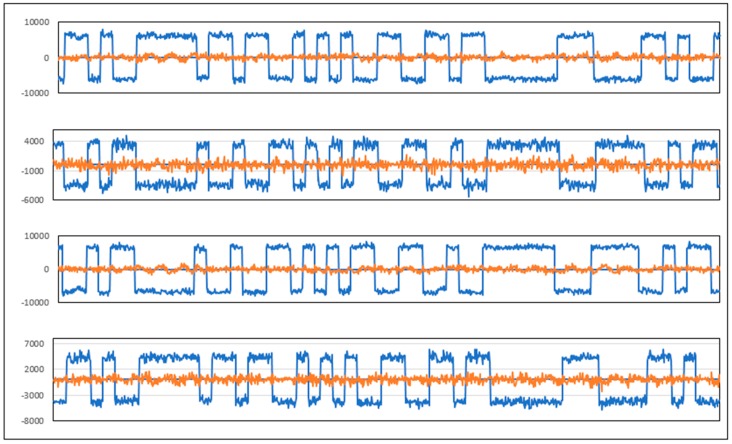
Prompt in-phase (blue) and quadrature (red) tracking results (for 1 s) of GPS C/A (coarse acquisition signal) multi-channel processing of real data using GNSS-ISE (SV12, SV15, SV24, SV32). Tracking is performed with 1 ms integration time, 20 ms per GPS C/A.

**Figure 17:**
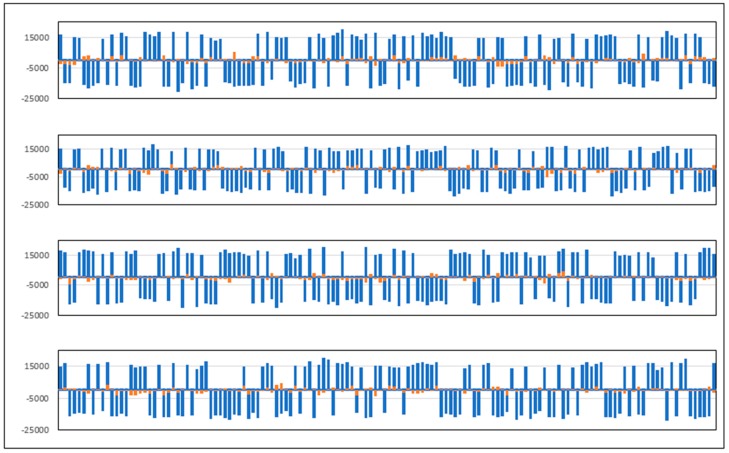
Prompt in-phase (blue) and quadrature (red) tracking results (for 500 ms) of GALILEO (European global satellite navigation system) E1B multi-channel processing of real data using GNSS-ISE (E02, E03, E05, E08). Tracking is performed with 4 ms integration time, 4 ms per GALILEO E1B bit.

**Figure 18:**
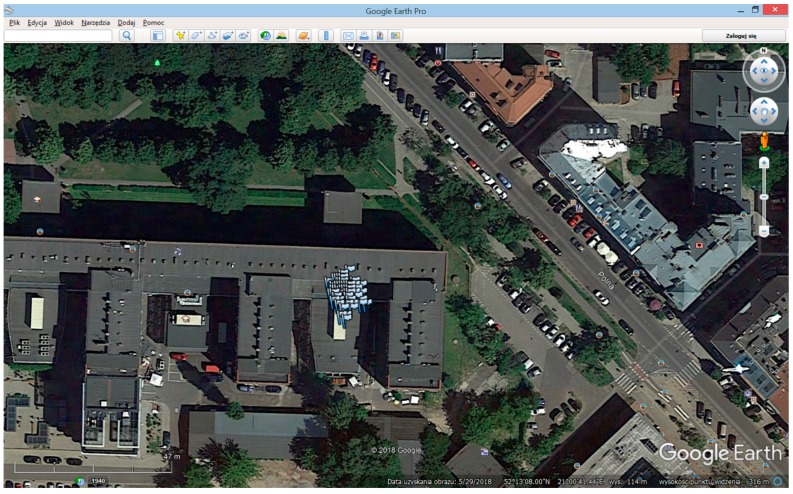
Position calculation using GNSS-ISE.

Figure 16 and Figure 17 present the tracking results of the GPS C/A and GALILEO E1B multi-channel processing of real data using GNSS-ISE. Figure 18 shows the early results of position calculation (see blue flags) using raw data obtained by the developed GNSS-ISE. The test was performed in stationary conditions with a GNSS antenna placed near the window edge. The antenna placement is sub-optimal but was conditioned by the measurement board connected to the measurement equipment. More robust positioning tests are in progress using stand-alone receivers with the CCNV1-A1 chipset. The purpose of this test was to prove the feasibility of implementing the GNSS-ISE instruction set extension into an embedded microprocessor. The other purpose was to prove the GNSS-ISE completeness in terms of delivering code measurements and carrier phase measurements, allowing effective post-processing.

Most of the works referenced in the introduction are focused only on one particular aspect of GNSS receiver architecture. Papers [11,12,13] describe the analog front-end only. In [16], the digital baseband was designed as an FPGA, while PVT computation was carried out by the PC software. In [20,21], the ASIP technique was used, but only PVT computation was under research and no real GNSS receiver results were shown. Work [17] presents the complete but not fully integrated classic architecture GNSS receiver implemented in a FPGA with an external analog front-end. On the other hand, this work presents the ASIP technique utilized to design the GNSS-ISE extension dedicated to GNSS baseband processing. The presented GNSS-ISE implementation result in the form of a fully integrated multi-constellation and multi-frequency single-chip GNSS receiver ASIC prompted authors of this work to provide comparison results only with other available ASIC implementations of GNSS receivers. There are only two other projects, according to the authors knowledge, that present an attempt to design a dedicated single-chip, multi-frequency, and multi-constellation ASIC [14,15]. None of them exploited the instruction set extension for GNSS baseband processing. Both approaches, involving a dedicated hardware engine on the system bus or presented in this work, GNSS-ISE, resulted in the implementation of a fully operational GNSS receiver. The authors do believe there are no disadvantages of using the GNSS-ISE. On the other hand, as stated before, the SDR approach facilitates the adaption of new modulation techniques or advanced tracking algorithms. Moreover, the tight connection to the CPU boosts the processing bandwidth, which is especially essential in a multicore system. Table 2 presents the comparison of the achieved parameters, provided in publications, of those two projects and the CCNV1-A1 chipset utilizing GNSS-ISE. It is worth noting that the presented CCNV1-A1 device is so far only one of the possible designs exploiting the GNSS-ISE. As can be seen, the achieved parameters are comparable. The die size is larger due to the lower technology node; however, the single tracking loop channel power consumption is significantly lower. The CCNV1-A1 provided a slightly lower number of tracking channels and a lower clock frequency due to the technology limitations, while it is the only one incorporating an embedded flash and additional application processor core. It is worth noting that the stress in the NaviSoC project was put into implementation of the widely market-available, low-cost, multi-frequency, and multi-constellation GNSS receiver. On the other hand, the main purpose of this work was to introduce a GNSS-ISE, a generic instruction set extension aimed for implementation of multi-frequency and multi-constellation GNSS receivers.

## 7. Conclusions

The authors presented the results of research toward designing the instruction set extension dedicated to GNSS baseband processing. Improved mechanisms for instruction set generations focused on multi-channel processing were introduced. The proposed method relies on the early identification of DAGs and parts of DAGs constituting the application’s CFG that violates the proposed $R_{univ}\left(B,a_{k}\right)$ relation. As a result, the developed ISE should easily adapt to new requirements. Identification of local parameters significantly reduces the number of DAG inputs and, consequently, improves the quality of the obtained results. Formatting the local parameters to custom register file windows ensures efficient multi-channel processing. The proposed approach can be used to generate instruction set extensions in other multi-channel-based wired or wireless data communication systems. The described GNSS-ISE extension was developed using the presented analytical approach for the generation of instruction set extensions. The ASIC implementation of multi-frequency and multi-constellation GNSS receivers incorporating the GNSS-ISE was performed. The fabricated CCNV1-A1 navigation processor consists of two analog front-ends for L1/E1 and L5/E5 bands, a three-core microcontroller with 32 GNSS-ISE channels, featuring a rich set of peripherals, 512 KiB SRAM, and 768 KiB eFlash. The presented results show the feasibility of implementing the GNSS-ISE into the embedded microprocessor system and its capability of performing baseband processing. Whereas the concept of exploiting a dedicated instruction set extension for GNSS baseband processing has not been previously presented in the literature, the presented comparison with state-of-the-art GNSS receivers shows that the GNSS-ISE can be successfully used to achieve comparable results. By adopting the SDR approach the embedded firmware gains flexibility in adapting to new modulation techniques and advanced tracking algorithms. This can be limited or even impossible using the traditional approach of designing GNSS receivers. The hardware tracking loops tightly coupled with the processor core boost the processing bandwidth essential in time-critical tasks. On the other hand, tracking loops arranged on the processors’ system bus have to share resources and access time between other peripherals and processor’ cores. The flexibility of GNSS-ISE allows a wide range of implementations, especially low-cost mass-market devices for IoT (internet of things) or smart sensor applications utilizing multi-frequency and multi-constellation GNSS data processing. According to the authors’ best knowledge, the developed GNSS-ISE has been incorporated into the first-in-the-world, integrated, multi-frequency, and multi-constellation microcontroller with embedded flash memory.

## Figures and Tables

**Table 1:** Summary of introduced Global Navigation Satellite System-instruction set extension (GNSS-ISE) instructions.

Instructions Group	Mnemonic
Channel Manipulation Instructions	gnss.chann.set gnss.chann.get gnss.chann.incr
Carrier Instructions	gnss.carr.freq gnss.carr.disc gnss.carr.set gnss.carr.rem
Accumulation Instructions	gnss.accu.add gnss.accu.get
Phase Lock Loop Instructions	gnss.pll.disc gnss.pll.costas gnss.pll.flt.rst gnss.pll.flt.coef gnss.pll.flt
Delayed Lock Loop Instructions	gnss.dll.disc gnss.dll.flt.rst gnss.dll.flt.coef gnss.dll.flt
Spreading Sequence Instructions	gnss.pcode.addr.set gnss.pcode.wr gnss.pcode.len gnss.scode.addr.set gnss.scode.wr gnss.scode.len gnss.code.get gnss.code.nco.freq gnss.code.epl.freq gnss.code.disc gnss.code.rng
Special Instructions	gnss.free.accu.wr gnss.free.accu.rd gnss.free.update.wr gnss.free.update.rd gnss.track.step

**Table 2:** Comparison with published multi-constellation and multi-frequency GNSS receivers.

Parameter	Values		
	[14]	[15]	This Work
Process	180 nm	65 nm	110 nm
ASIC size	n/a	4.5 × 5.0 mm	5.7 × 6.0 mm
Power	n/a	12.7 mW/channel	5 mW/channel
Tracking channels	36	40	32
Processor cores	1	1	3
Application processor	no	no	yes
Processor frequency	90 MHz	150 MHz	80 MHz
GNSS frequency	up to 50 MHz	up to 75 MHz	up to 65 MHz
FFT module	128 points	16k points	2 × 256 points
RF front-end	no	yes	yes
Embedded flash	no	no	768 KiB
Internal SRAM	no	1 MiB	512 KiB
Target market	space	research only	low-cost consumer
